# Vitamin D Supplementation, Characteristics of Mastication, and Parent-Supervised Toothbrushing as Crucial Factors in the Prevention of Caries in 12- to 36-Month-Old Children

**DOI:** 10.3390/nu14204358

**Published:** 2022-10-18

**Authors:** Piotr Sobiech, Dorota Olczak-Kowalczyk, Marie Therese Hosey, Dariusz Gozdowski, Anna Turska-Szybka

**Affiliations:** 1 Department of Paediatric Dentistry, Binieckiego 6, Medical University of Warsaw, 02-097 Warsaw, Poland; 2 Centre of Oral, Clinical and Translational Sciences, Faculty of Dentistry, Oral and Craniofacial Science, King’s College, London WC2R 2LS, UK; 3 Department of Experimental Design and Bioinformatics, Faculty of Agriculture and Biology, Warsaw University of Life Sciences, Nowoursynowska 166 ST., 02-787 Warsaw, Poland

**Keywords:** mastication, diet, oral hygiene, parent-supervised toothbrushing (PSB), resilient food, severe early childhood caries, vitamin D supplementation

## Abstract

Severe early childhood caries (S-ECC), defined as any sign of smooth-surface caries in a child younger than three years of age, remains a serious health issue. The aim of this study was to indicate oral health behaviours related to S-ECC. The study surveyed parents (socio-economic and medical factors, oral health behaviours) and clinically examined children including non-cavitated d1,2/cavitated d ≥ 3 lesions. %S-ECC, and caries indices (d1,2 d ≥ 3 mft and d1,2 d ≥ 3 mfs) were calculated. Spearman’s correlation and simple and multiple logistic regression were used to assess the relationships between various factors and S-ECC. A total of 496 children were examined. S-ECC occurred in 44.8%: d1,2 d ≥ 3 mft = 2.62 ± 3.88, d1,2 d ≥ 3 mfs = 4.46 ± 8.42. S-ECC was correlated with socio-economic factors, vitamin D supplementation, breastfeeding and using formula after the 18th month, and toothbrushing. Supplementation of vitamin D and toothbrushing tended to decrease the odds of S-ECC (OR = 0.49 (0.27–0.87); *p* = 0.016, OR = 0.46 (0.24–0.86) *p* = 0.015, respectively). Feeding exclusively with formula was observed to increase the odds of S-ECC (OR = 2.20 (1.29–3.76); *p* = 0.004). Consuming > three snacks daily (OR = 1.39 (0.97–1.98); *p* = 0.072) and the reluctance to eat resilient foods (OR = 1.63 (1.05–2.51); *p* = 0.028) were nullified by the confounding factors. Vitamin D supplementation, mastication of resilient food, breastfeeding in the first six months of a baby’s life, and parent-supervised toothbrushing are factors in the prevention of caries in toddlers. Breast- and bottle-feeding after the 18th month of life, the reluctance to eat solids, lack of vitamin D supplementation, hygienic neglect, and delay in introducing oral health behaviours may contribute to the development of caries in toddlers.

## 1. Introduction

Dental caries in very young children, i.e., developing soon after tooth eruption, has particularly poor prognosis. Insufficient tissue mineralization of newly erupted teeth promotes rapid spread of the disease. Tooth decay in very young children usually progresses rampantly. For this reason, every smooth-surface carious lesion in a child aged < 3 years is defined as severe early childhood caries (S-ECC). S-ECC can also be diagnosed in children at the age of 3 years (when carious lesions are found on at least four dental surfaces), 4 years (five dental surfaces), or 5 years (six and more dental surfaces) [1]. Opposite to S-ECC, early childhood caries (ECC) is defined as the presence of one tooth or more affected by dental caries, extracted (missing) or filled due to a carious lesion in children aged 71 months (5 years and 11 months) [1].

S-ECC impacts the oral health-related quality of life (OHRQoL) of a child, their general development, and proper development of the masticatory apparatus, and thus plays a role in establishing proper social interactions and the health of the body [2,3,4,5]. The presence of this disease is costly economically, socially, and health-wise. Decay of deciduous teeth may cause pain and local periodontal infections which may spread systemically; its management may necessitate root canal treatment, extractions, antibiotic therapy, and hospitalization [1,2]. It hinders development of speech and biting and chewing of food [4]. It has been demonstrated that there is an association between the presence of teeth with extensive carious lesions and decreased increment of body mass, iron deficiency anaemia, and sleep disruption [6]. The decay of deciduous incisors in particular affects the aesthetics, which hinders the development of interpersonal communication [2,3]. 

Unfortunately, it affects about 621 million children worldwide from 11.7% in England to 41.1% in Poland and 46.8% in the USA [7,8,9]. 

A number of researchers have analysed the significance of various associated factors of ECC [1,2,4,10,11]. On the basis of these results, it was possible to identify a variety of environmental factors, including parental attitudes and pro-health activities concerning the oral cavity, as well as demographic, socio-economic, and medical factors that may increase the odds of caries in deciduous teeth. Proven significant behaviours include excessive exposure to sugar, too many meals (more than 4–5), frequent snacking, night feeding after one year of life, and bottle nursing after the 18th–24th month of life. Other influencing factors have also been suggested, these are: neglect of hygiene, absence of fluoridation prophylaxis, enamel hypoplasia, vitamin D insufficiency, and tobacco smoking by mothers, as well as medical factors such as systemic diseases, C-section or premature delivery, and low birth weight [2,4,6,9,10,11,12,13,14]. Most of the studies were conducted on older children. 

Few researchers have evaluated health behaviours from the S-ECC perspective in children in the first three years of life, i.e., in the period when dental caries has its onset. What is more, studies assessing dietary patterns of very young children focused on the cariogenicity of foodstuffs and the frequency of their consumption by children. The significance of introducing solids to the child’s diet, a child’s preference of the consistency of food, performing hygienic practices in the child’s mouth within the time such practices should be initiated, or vitamin D supplementation is still unknown. The importance of mastication, consumption of resilient foodstuffs that require chewing, and vitamin D supplementation have not been sufficiently studied in the group of the youngest children. 

The aim of the study was to report on the oral health behaviours that are related to the incidence and severity of S-ECC in children aged from 12 to 36 months of life.

We hypothesized that parent-reported vitamin D supplementation, diets rich in solids requiring chewing, parent-supervised toothbrushing (PSTB), and early initiation of such practices would be related to lower caries experience in children assessed by dental caries scores (d1,2 d ≥ 3 mft/d1,2 d ≥ 3 mfs indices). 

## 2. Materials and Methods

### 2.1. Study Population

A sample size determination (minimal required sample size) was performed using a simple ‘survey’ calculator (available on: http://www.raosoft.com/samplesize.html accessed on 15 February 2009), assuming the population of 12- to 36-month-old children in Warsaw at about 50,000 and caries prevalence at about 40%. This determined that 450 participants (assuming 25% redundancy) would need to be recruited to achieve the margin of error not greater than 5% for binomial proportion for children with caries at a 95% confidence level. 

This cross-sectional study comprised clinical examination of children’s dentition and questionnaires filled in by a parent/legal caregiver. The study was part of the 2011–2017 programme assessing oral health and the teething process. The consent of the Bioethics Committee of the Medical University of Warsaw was obtained (KB/221/2009). The invitation to participate in the study was placed in periodicals on the subject of maternity, as well as in crèches and clinics for children. The inclusion criteria were: the child’s age of 12–36 months, residence in the city of Warsaw or its suburbs within a 20 km radius (the level of fluoride in the drinking water <0.3 mg/L), signed informed consent for the child’s participation in the clinical examination and for parental survey and a fully answered questionnaire, and child’s co-operation enabling the clinical examination. Exclusion criteria were: intellectual disability, chronic systemic disease, and those taking medication that potentially affected oral health (e.g., pH-lowering drugs, drugs that decreased salivary flow and buffering). 

Those for whom there was no written consent from a parent/caregiver or those who returned an incomplete questionnaire were also excluded. 

The enrolled children were assigned to four age groups: 12–18 months, >18–24 months, >24–30 months, and >30–36 months.

### 2.2. Clinical Examination 

The clinical examination of children’s dentition was carried out in a dental office with shadowless lamp illumination using a dental mirror and a WHO-621 periodontal probe. In the assessment of the condition of teeth the following were recorded: the number of erupted teeth, the presence of carious lesions and fillings, and the number of missing teeth. All dental surfaces in subsequent quadrants were examined. Carious lesions were assessed in accordance with the modified International Caries Detection and Assessment System—ICDAS II [15]. Dental caries was identified as a non-cavitated or cavitated lesions in a child in the first three years of life, in accordance with the definition of severe early childhood caries (S-ECC) for children aged < 3 years [1]. 

The following were calculated: (i) the incidence and (ii) severity of dental caries in the youngest children. The prevalence of S-ECC is the proportion of individuals with non-cavitated and cavitated caries, classified as affected (S-ECC > 0 or d1,2 d ≥ 3 mft > 0 and d1,2 d ≥ 3 mfs > 0). A recording of “d1,2” represented non-cavitated carious lesions (Code 1 and 2 acc. to ICDAS II), “d ≥ 3”—cavitated carious lesions (Codes 3 and higher acc. to ICDAS II), “m”—missing due to caries, “f”—filled due to caries.

Decayed (d), missing (m), and filled (f) teeth (t)—(dmft) indices and decayed (d), missing (m), and filled (f) surfaces (s)—(dmfs) indices, reported in this study as the d1,2 d ≥ 3 mft and d1,2 d ≥ 3 mfs indices for assessing dental caries severity, are used.

The examinations were conducted by three experienced dental practitioners with specializations in pedodontics, following training and calibration. Assessment of the consistency of each individual examiner (intra-examiner reproducibility) and also the variations between examiners (inter-examiner reproducibility) were performed. Each paediatric dentist (examiner) independently examined the same group of ten patients, and the findings were compared with those of the experienced supervisor. Cohen’s Kappa coefficient between the reference dentist (DO-K) and the other dentists was between 0.89 and 0.95 for carious teeth and should be interpreted as an almost perfect agreement.

### 2.3. The Collection of Socio-Demographic and Lifestyle Data 

The questionnaire for the parent/legal caregiver covered the following areas of interest: (i) socio-economic factors (level of education and the parent’s age, self-assessment of the family’s economic status); (ii) medical factors (chronic diseases of the mother, course of the gestation period, infant’s perinatal parameters); (iii) child’s feeding patterns (breast/bottle-feeding and the age when this type of feeding was terminated, duration of breastfeeding (in the first six months of life; >12th month of life; >18th month of life), bottle-feeding with formula (>12th month of life; bottle-feeding with formula >18th month of life)), age at which solids were introduced, consumption of solids, daily frequency of consumption of solids, the current number of meals and snacks during the day, child’s willingness to consume foodstuffs that require chewing, addition of sugar to individual dishes, offering fruit as snacks; (iv) child’s hygienic and preventative practices (age of introducing hygienic practices, frequency of toothbrushing, and the age it was started, adult-supervised toothbrushing, the use of fluoride-containing toothpaste, endogenous fluoride prophylaxis, and parent-reported vitamin D supplementation, following Polish recommendations), topical fluoride prophylaxis, and dental check-ups (the first visit).

In Poland, the legally determined 5-level classification of education includes: primary (elementary), vocational, secondary, post-secondary, and high. The authors decided to form three categories in the following fashion: 1—primary and vocational, 2—secondary and post-secondary, and 3—higher. The respondents failed to reveal their monthly household income, and so the economic status criterion was subdivided into three levels (high, middle, and low) depending on one variable—respondent’s subjective evaluation.

The questionnaire was designed by the principal investigator (D.O-K.) and based on the relevant literature concerning oral health behaviours related to ECC and brief food frequency questionnaire (FFQ) [7,9,10,11,12,13,14,16,17]. The questionnaire was pre-tested and validated twice on a group of twenty parents of children presenting for check-ups. 

### 2.4. Statistical Analysis

To compare mean values of severity of S-ECC between two groups distinguished based on pro-health, hygienic, and nutritional behaviours, e.g., with and without vitamin D supplementation, a *t* test was carried out. Spearman’s correlations were used for evaluation of the relationships for the selected pairs of variables to evaluate relationships between socio-economic factors and oral health behaviours with the occurrence and severity of caries.

In statistical analysis logistic simple and multiple regression was used for evaluation of various pro-health, hygienic, and nutritional behaviours on the incidence of S-ECC. On the basis of these analyses, odds ratio (OR) and adjusted odds ratio (AOR) with confidence intervals (CI) at the confidence level of 95% were specified. The confounding factors were: for AOR-1—socio-economic factors (self-assessed economic status, education level of parents), for AOR-2—hygienic behaviours (frequency of toothbrushing, using fluoride toothpaste), for AOR-3—dietary behaviours (vitamin D supplementation > 12th month of life, more than three snacks a day, reluctance to consume foods that require chewing), for AOR-4—socio-economic, hygienic, and dietary factors. The confounding factors were defined as those that are statistically significant for dental caries in Spearman’s analysis. In all the analyses, the significance level was set at *p* < 0.05. Statistica 13 was used to perform relevant analyses.

## 3. Results

Of the 827 children who had declared their participation in the study, a total of 496 children who completed the exams and were considered eligible for this analysis were enrolled (mean age 24.16 ± 6.93 months), with 262 (52.8%) boys. For data analysis, children were divided into four age groups: 12–18 months; >18–24 months; >24–30 months; >30–36 months (Table 1). The number of subjects in each group varied, ranging from 104 to 135. The reasons for exclusion of the 331 children were: incorrectly completed questionnaire (13), younger than 12 months (248), and older than 36 months (70). 

All the questionnaire forms were filled in by the mothers of the examined children. The socio-economic characteristics of both parents and the medical data of the mother are presented in Table 2.

The most common chronic diseases reported by mothers included diabetes (*n* = 17), hormonal imbalance (*n* = 31), and allergies (*n* = 47). During pregnancy, the most common health issues were diabetes (*n* = 28), anaemia (*n* = 30), and cardiovascular diseases (*n* = 21). For five children (1.0%), the gestation period lasted <28 weeks. A birth weight <2500 g was confirmed in 32 children (6.5%). The mean gestation period was 38.88 ± 2.29 weeks, and the mean birth weight was –3366.44 ± 561.59 g. Most children (333—67.1%) were born via natural delivery. 

Table 3 presents information on dietary and hygienic behaviours of children perceived as the most important and significant following further analysis. 

In the first six months of life, 257 children (58.1%) were mix-fed, i.e., breast-nursed and bottle-nursed with formula. All the respondents confirmed that they would add sugar to dishes and beverages consumed by a child from the second year of life. The mean age when the child’s diet began to be supplemented with any food other than milk was 5.40 ± 2.10 months, feeding with a spoon—6.12 ± 2.57 months, and introduction of solids—7.58 ± 3.02 months. Every fifth mother reported that her child was reluctant to chew resilient foods; therefore, they were fragmented before giving them to a child, or replaced with soft foods. The mean number of meals consumed by a child (main meals and snacks) was 5.65 ± 1.07.

Forty-five (9.1%) mothers reported that they did not clean their child’s teeth at least once a day, and seventy-nine (15.9%) claimed that they began doing this only in the second year of the child’s life. The age of children when toothbrushing was started ranged from 4 to 28 months (mean age 10.67 ± 4.31 months). None of the children had ever received fluoride tablets or drops. Mothers reported that they used toothpaste “suitable for the child’s age.” Non-fluoridated toothpaste was used by 22 (4.9%) respondents. All the children in the first year of life were given vitamin D; however, after the first year of life supplementation was continued in 86.3% of cases.

In the whole examined sample, S-ECC was diagnosed in 44.8% of children. Thus, S-ECC was present in every fifth child in the youngest age group and in more than half of the oldest children (Table 4); d1,2 d ≥ 3 mft and d1,2 d ≥ 3 mfs indices increased with age and the number of erupted teeth. 

Spearman’s rank correlation analysis that assessed parameters related to dental caries in a child did not confirm the significance of the course of pregnancy or perinatal parameters of an infant for the occurrence and severity of S-ECC. Statistically significant correlation between maternal chronic diseases and d1,2 d ≥ 3 mfs in a child was revealed only in the >18—24 months age group (r = 0.187). The impact of diabetes during pregnancy was significant in the whole sample; however, the values of Spearman’s coefficients were only 0.092 for d1,2 d ≥ 3 mfs and 0.090 for d1,2 d ≥ 3 mft > 0. Spearman’s rank correlation coefficients indicating socio-economic factors and oral health behaviours statistically significantly related to the occurrence and severity of caries in children in the whole studied sample and the age groups individually are presented in Table 5. Statistically significant factors were: the level of education and parental age, self-assessed economic status of the family, hygienic and dietary behaviours.

Links between dietary and hygienic factors and the occurrence and severity of caries in a child in individual age groups were found. No statistically significant correlation between caries and the moment when foods other than milk were introduced was found in the whole examined sample. The same can be said of the moment when the children started to be fed with a spoon and of the number of fruity snacks. The number of fruity snacks only mattered in the youngest age group (r d1,2 d ≥ 3 mfs = 0.245, r d1,2 d ≥ 3 mft > 0 = 0.235). 

Logistic regression analysis that was carried out for the whole sample confirmed significantly lower incidence and severity of caries in children who were supplemented with vitamin D after the first year of life, and lower incidence of caries in children whose teeth were brushed (Table 6 and Table S1). 

Greater odds of developing caries were observed when: first—a child was not breastfed in the first six months of life, second—there were too many meals after the 12th month of life, and third—a reluctance to chew solid food was observed. The significance of vitamin D supplementation, frequency of meals, and reluctance to chew were decreased by socio-economic factors. None of the confounding factors significantly changed the importance of toothbrushing and bottle-feeding the child with the formula only. 

## 4. Discussion

The presented results confirm the significance of socio-economic factors in the aetiology of ECC and identify oral health behaviours that increase the odds of the occurrence of caries in toddlers residing in areas with low fluoride content in drinking water, and in those who consumed foods and beverages with sugar as early as in the first two years of their life. As is commonly known, exposure to sugar in the first two years of life is one of the fundamental factors contributing to the development of highly cariogenic biofilm, increasing the risk of ECC [2,4]. According to WHO [18] recommendations, sugar should not be added to foods and beverages that a child consumes in the first two years of life. Yet, studies that assess the consumption of sugars in 18 countries indicate disproportionately high consumption in childhood. Total sugar content as energy percentage is high in childhood, the highest in infants and children aged < 4 years [18]. Epidemiological studies conducted in Poland revealed that sugar was added to 86.7% of dishes and beverages consumed by children in their first two years of life, which increased the risk of them developing ECC at the age of 3 years [10].

The observations of the authors of this study confirm the influence of a child’s dietary pattern, both in the past and the present, on the occurrence and severity of caries in deciduous teeth. Health benefits, both for a child and their mother, are undoubtedly brought about by breastfeeding, especially in the first six months of a child’s life. Children who are exclusively fed with infant formula had a two-fold chance of developing dental caries. This association emerged regardless of the type of confounding factors that were being introduced to the statistical model. The severity of caries was also twice as high. The beneficial effect of breastfeeding in the period preceding teeth eruption also leaves no doubt. After the first teeth erupt, their cleaning is a necessary practice, and also after breastfeeding [19]. It has been demonstrated that human milk may, to a certain degree, promote development of caries, especially in the presence of sugar. In the period between the 3rd and 9th month after delivery, mother’s milk may support the formation of *S. mutans* biofilm [20]. Several studies indicate that breastfeeding does not decrease the pH of biofilm, regardless of the condition of children’s teeth [2]. Brazilian researchers proved that human milk has cariogenic potential, even higher than cow’s milk, yet considerably lower than sucrose. They also emphasized that the addition of sucrose to human milk exacerbates demineralization. Thus, prolonged breastfeeding can unfavourably affect children exposed to other cariogenic foods and beverages [21]. If human milk were the only source of carbohydrates in a child’s diet, it would not be cariogenic.

In the examined sample in the present study, the incidence and severity of caries in children breastfed and bottle-fed with formula after the 18th month of life increased in comparison with children fed for shorter periods. These differences were not statistically significant. What was significant was Spearman’s rank correlation illustrating the association between dental caries and breastfeeding or bottle-feeding after the 18th month of life.

Still debatable is the issue of breastfeeding after the second year of life. Surveys among Polish parents/legal caregivers of children aged 3 years, and clinical examinations of a nationally representative sample of 3-year-olds living in all 16 provinces of Poland, revealed that the very fact of breastfeeding, or the age at which it was terminated, should not be treated as the risk factor of ECC [10].

The meta-analysis of 35 studies covering a total of 73,401 children aged 0–71 months revealed that breastfed children ran a smaller risk of developing ECC in comparison with their peers who were only bottle-fed (OR = 0.77, 95% CI: 0.61–0.97, *p* = 0.026) [22]. These results tally with observations expressed in this study. In the examined sample, bottle-feeding with formula promoted the occurrence and severity of dental caries whereas breastfeeding only was negatively correlated with d1,2 d ≥ 3 mfs. The above-mentioned meta-analysis indicated that breastfeeding after the 12th month of life significantly increased the risk of ECC in comparison with children who were breastfed in their first year of life only (OR = 1.86, 95% CI: 1.37–2.52, *p* < 0.001). In the present study, in the 18–36 months’ age group, both prolonged breastfeeding and bottle-feeding favoured dental caries.

The type of milk given to infants matters, and its effect varies. Feeding an infant with formula adversely affects the shaping of taste preferences of a child, which may also be related to the occurrence of caries. Breastfed children of mothers who vary their diet will more eagerly try new dishes. The examination of children aged 2 to 8 years also indicated the significance of the breastfeeding period. Naturally, in order to achieve this positive influence of breastfeeding on a child’s dietary preferences, the mother has to enjoy a varied diet during the lactation period, which will produce a varied taste profile of her milk. Formula-fed infants are not exposed to taste variations of mother’s milk. They become used to the taste of formula and may find it difficult to accept other tastes, for example fruits and vegetables [23].

Introduction of additional foodstuffs after the first six months of infancy is particularly important in the shaping of dietary behaviours. In this period, newly introduced food should be less and less fragmented, and the child should become acquainted with its sensory (consistency, taste, and aroma) and nutritious aspects (energetic density) [23]. This period is fundamental because introduction of dietary changes in the second year of life plays a lesser role in the shaping of dietary behaviours. Children aged 2–5 years have a heightened level of dietary neophobia, i.e., they refuse to consume “new, unfamiliar” food. It has been observed that the willingness to consume new dishes is mostly determined genetically and modified environmentally and culturally [23]. Giving a child sweetened food to make it more acceptable in infancy and thereafter forms a belief that food should generally be sweet. This is an important observation, as it is a frequently quoted reason for the difficulty in controlling a child’s diet [24].

Solids not only stimulate biting and chewing but also increase salivation. As consecutive deciduous teeth erupt, parents should attempt to change the consistency of food and gradually replace liquid and sticky foods with those that are harder to chew to stimulate mastication and prevent the so-called “lazy chewing”. In the sample examined in this study, the age when solids were introduced was positively correlated with the occurrence and severity of caries, and this correlation was particularly apparent in children >18–24 months of age. In the same age group, there was also a marked correlation between caries and reluctance to eat food that required chewing, resulting in its fragmentation or replacement with softer dishes, sometimes sweetened. Eating soft consistency dishes and poor chewing will weaken the mechanism of oral self-cleansing and may decrease the secretion of saliva. It has been demonstrated that the very act of chewing, without the taste factor, will stimulate salivary glands, yet to a lesser extent than the gustatory experience, especially if it is sour.

Children as young as three years are relatively capable of performing the function of chewing, just like adults. Foodstuffs which have increased values of hardness are regarded as beneficial since their chewing requires more muscle effort, which in turn will positively correlate with the child’s growth and development. It is common knowledge that the development of dental caries is influenced by the hardness of food [25]. Hard and coarse foods may exert a detergent effect during chewing, which is effective in preventing caries. It is believed that the soft diet of today lacks physical properties which require more energetic chewing; this makes teeth more susceptible to caries [26]. Many foods and their constituents have an anticaries potential [27]. Recent studies have indicated that fruits, cheese, and fibre-rich foodstuffs may diminish the activity of metabolic acids and help restore enamel which is lost during chewing [28]. Advocated restorative mechanisms include buffering, stimulation of salivary secretion, even higher reduction in bacterial adhesion, and decreasing enamel demineralization and/or promoting remineralization with casein, calcium, and phosphorus. Comprehensive information on foods that have a cariostatic potential can be found in the 1970–2014 review by Sandhu et al. [29]. Fruits contain fibre and polyphenols, which have a potential to disrupt the formation of bacterial plaque and can inhibit acidogenicity of oral bacteria [27]. The total index of healthy nutrition may reflect the general nutritional pattern in which fruits, grain, and dairy products are frequently consumed, superseding processed food which has added sugar and sodium. Studies by Nunn [30] and Zaki et al. [31] indicate a significant correlation between complying with general guidelines for healthy nutrition and a decreased risk of developing S-ECC in toddlers. The frequency of consumption of fresh fruit was higher in caries-free individuals in the study by Bahugun et al. [32]. They concluded that fresh fruit does not stay in the mouth too long, and additionally contains fructose which is less cariogenic than sucrose. Some plant foods are a good source of flavonoids (phenolic compounds and organic acids) which demonstrate antimicrobial action and may also have a cariostatic property. Fibrous plant foods are sometimes perceived as natural toothbrushes; their efficiency in preventing caries, however, is rather related to the mechanical stimulation of salivary flow than the effective removal of dental plaque. Flavonoid compounds have antiadhesive properties, and phenol compounds damage bacterial cell walls, which is partly reflected in their antibacterial effect. Clinical studies on the effectiveness of fruit, and apples in particular, in prevention of caries are not consistent, and their results are ambiguous. Apples contain sugars and malic acid, and their consumption is a mechanical booster for salivary flow. Consumption of fewer than five portions of fruits and vegetables a day translates into higher caries experience in children aged 2–5 years [33]. Edwards et al. from Trinidad postulate that consumption of fruits between meals does not impact caries development. Conversely, significant factors affecting a high experience of dental caries are consumption of fresh fruits and non-alcoholic beverages [34,35]. In the present study, in the youngest age group (12–18 months), however, an unexpected, unfavourable association between caries and fruit consumed as snacks was revealed in accordance with the previously cited results. It is possible that the fruit was mashed or processed. Unfortunately, this study failed to specify the form in which the fruit was given to a child, which is the study’s limitation, hence the need to design a new study which will account for this oversight.

In the literature, one can find favourable opinions on how consumption of hard foodstuffs plays a role in the reduction in the caries severity. According to reports, solid consistency of food stimulates mastication, which assists in natural cleansing of the oral cavity from food debris through the intense movement of cheeks, lips, and the tongue. This way, the risk of caries is decreased. Additionally, hard foodstuffs and mastication itself signal increased salivary secretion, which is supposed to facilitate the formation of the food bolus. In the studied sample, reluctance to chew was responsible for the 1.63-fold increase in the occurrence of caries. In children who were unwilling to consume resilient foods, the d1,2 d ≥ 3 mfs values were statistically significantly higher. Another interesting observation is that hygienic confounding factors introduced to the statistic model did not alter the negative influence of avoiding foods that require chewing. Yet, it was nullified by the dietary factors.

The risk of caries in a child was also increased by frequent consumption of meals; however, the introduction of dietary confounding factors produced a statistically significant reduction in this risk. Studies on 3-year-olds revealed the significance of not only the frequency of food/beverage consumption, but also their kind. Quenching thirst with water or unsweetened milk, as well as preference for healthy snacks (sandwiches, fresh fruit), lowered the risk of caries, even if other, improper dietary behaviours or hygienic practices concurred [28].

The presented results illustrate the complexity of the significance of diet in the aetiology of dental caries. They also confirm the importance of what happens in the first year of life in the shaping of a child’s dietary behaviours and the health of their dentition.

Our studies revealed that brushing a child’s teeth by an adult and parent-supervised toothbrushing (PSB) are fundamental factors. According to the AAPD, parents should aid their children in toothbrushing, since younger children lack motivation and the dexterity required to brush their teeth [1]. PSB involves supervision of brushing the teeth twice a day with fluoride toothpaste upon the eruption of the first tooth to at least 7 years of age. This is a dyadic process in which children allow their teeth to be brushed by parents. Significant associations were found between dental caries and PSB [36,37,38]. Parental behaviours toward their child’s oral health including assistance in toothbrushing and motivation to brush twice daily were found to be protective against ECC [39]. The age of children when they start to brush, not brushing regularly, and the presence or absence of parental assistance were also significantly related to ECC [39]. Starting to brush at an earlier age, parent-supervised brushing, and increased frequency of toothbrushing can often reduce the risk of caries, which is consistent with the results of some studies [39]. Parental assistance improved the quality of oral hygiene procedures [40]. In the present study, only 9.1% of mothers admitted that they did not clean their child’s teeth at least once a day. In a study by Sankeshwari et al. [40], parental assistance in oral hygiene was not reported in 37.27%, and 67.22% of these suffered from ECC. Despite the fact that some parents are not aware of the guidelines for supervised brushing, they have knowledge of children’s brushing practices, brush children’s teeth independently twice a day as part of the family routine, and remind children to brush [37,38,41].

The study of Elison et al. [41] described the various perceived barriers and a variety of challenging situations that mothers face to brush their child’s teeth. Regrettably, toothbrushing seldom begins soon after their eruption on account of absence of co-operation from the child. Among the excuses for hygienic neglect, parents reported child’s resistance, temper tantrums, teething-related pain, and even child’s fatigue. In the present sample, the mean age of the initiation of the brushing habit was higher than the eruption of the first tooth. Seventy-nine (15.9%) claimed that they began brushing only in the second year of the child’s life. The age of children when toothbrushing was started ranged from 4 to 28 months (mean age 10.67 ± 4.31 months).

In the present study, fluoridated toothpaste was used by 94.9% respondents, similarly the findings of to Sankeshwari et al. [40]. Almost 95% of the children in the Sankeshwari et al. [40] study used toothpaste to clean their teeth. Although not much of a difference was seen between those who brushed once and those who brushed often, a large difference was seen between those who brushed and those who did not.

The use of fluoride-containing toothpaste should begin with the emergence of the first deciduous tooth [2,19]. Not every parent cleaned their child’s teeth, and some used a non-fluoridated toothpaste. Milgrom et al. [6] made a similar observation and claimed that 81% of parents cleaned their children’s teeth, of which 74% used a brush and 69% used toothpaste, which in 49% of cases contained fluoride, at the age of 12 months. Authors emphasized the significance of the use of fluoride varnish and toothpastes with more than 1000 ppm of fluoride in the prevention of caries, yet its role seems minor in comparison with dietary behaviours [28,42]. In 3-year-olds, brushing teeth twice a day lowered the risk of caries, but the effect was markedly overshadowed when improper dietary behaviours were considered [28]. Not knowing the fluoride content of the toothpaste used is certainly a limitation of the present study. Mothers failed to provide this information because of their insufficient knowledge. According to other Polish studies conducted among parents of 1–6-year-olds residing in Warsaw, 90% of children used toothpaste containing 500 ppm F, 4%—1000 ppm F, and 6% chose non-fluoridated toothpaste [43].

An important aspect of proper nutrition of children is supplementation of nutrients, if their supply in food is insufficient to cover the daily requirements of a small child. The main focus is on vitamin D as related to rickets prophylaxis. Continued supplementation with vitamin D of children at least in the autumn–winter period after 12th month of life was responsible for a two-fold decrease in the risk of caries or its exacerbation. The beneficial effect of vitamin D supplementation in this study was not changed by either dietary or hygienic behaviours.

Many studies assessed the association between vitamin D and dental caries [42,43,44,45,46,47,48,49].

The highest level of evidence on the impact of vitamin D and dental caries in children has been provided by Hujoel [43], who conducted a systematic review and meta-analysis of controlled clinical trials assessing the role of vitamin D on prevention of dental caries. Wagner and Heinrich-Weltzien [50] stated that no vitamin D supplementation (OR = 1.9 and CI 0.99–3.51) promoted caries, and the caries risk assessment forms needed to include vitamin D supplementation. Reviews and meta-analyses of Theodoratou et al. [44] established that the correlation between vitamin D and dental caries in children was probable, however, further analysis was needed. Some studies did not confirm such correlations [51,52].

The main form of vitamin D in the blood is 25-hydroxyvitamin D (25(OH)D), and its concentration provides a reliable indication of vitamin D levels. Children with severe early childhood caries (S-ECC) had lower concentrations of 25(OH)D and had twice the odds of having inadequate levels (less than 75 nmol/L) compared with caries-free children [47]. The authors of the present study did not check the 25(OH)D level in blood, but vitamin D supplementation only.

Some mechanisms of action may explain the role of vitamin D in reducing caries risk, e.g., the regulation of calcium and phosphate, which are important in formation, calcification, and mineralization of teeth [43,44]. Calcification of primary teeth starts in the second trimester of pregnancy and is continued after child’s birth. Based on studies on vitamin D and vitamin D receptors, vitamin D contributes to enamel and dentine formation and maturation [45]. The immunological role of vitamin D lies in inducing the formation of antimicrobial peptides (AMPs), such as the cathelicidins, the α- and β-defensins, and peptides with a high proportion of specific amino acids (the histatins), which are active and effective against oral cariogenic microorganisms [43,44,45]. Salivary antimicrobial peptides concentrations are higher among caries-free children. Vitamin D prevents infection by regulating B-cell proliferation and immunoglobulin production. The properties of saliva are important in remineralization–demineralization dynamic processes. “Vitamin D is essential for the maintenance and utilization of a specific pool of calcium required for normal fluid and electrolyte of the saliva of the parotid gland. Low salivary flow rate and high viscosity are recognized factors regarding high caries risk” [53].

According to results of systematic reviews and meta-analyses of controlled clinical trials that assess the impact of vitamin D on caries prevention, its supplementation may decrease the risk of caries development by 47% (95% CI: 35, 60, *p* < 0.0001) [43].

Vitamin D deficiency, actually, is accompanied by insufficient activity of antibacterial peptides, i.e., cathelicidins and defensins, decreased saliva secretion, and a low level of calcium in saliva [14,43,45]. The geographical position of Poland, resulting in insufficient exposure to the sun, compounded by insufficient time spent outdoors, the use of solar protection, and a diet poor in vitamin D, all contribute to high risk of vitamin D deficiency in the Polish population [14]. It has been demonstrated that in 80–95% of Polish children aged 1–3 years, their diet does not cover the daily requirement for vitamin D, hence the need to supplement [14]. Therefore, in Poland, and in other European countries, it is recommended that children aged 1–18 years take 10 µg/day of vitamin D from October to March, or throughout the whole year, if in summer months the amount of vitamin D synthesized by the skin remains insufficient [14,45]. Studies carried out in Poland on a group of 1638 children aged 3 years revealed that 99.1% of infants were supplemented with vitamin D; however, after the 12th month of life supplementation was continued seasonally only in 55.2% of children [14]. The studies referenced here also revealed that the incidence of ECC/S-ECC and mean dmft/dmfs indices in children who received vitamin D were lower in comparison with their non-supplemented peers. It was, noted, however, that the addition of confounding factors to the statistic model, such as the level of mother’s education, nullified its importance [14].

The attitude of parents concerning a child’s oral health plays a fundamental role and is related to socio-economic factors such as the level of education, among others. The present studies confirmed this thesis. It has to be stressed that the introduction of socio-economic elements as confounding factors to the logistic regression model modified the role of vitamin D supplementation and dietary behaviours. It is worth stressing that the Spearman’s rank correlation indicated the significance of the education level and the age of both parents. It emphasizes the current parental role in maintaining oral health of their offspring.

The present study had a number of identified limitations. The first is the cross-sectional study design. The cross-sectional nature did not allow us to indicate any causality of the examined factors between S-ECC and other related factors. Lifestyle behaviours such as the dietary intake could not reflect longitudinal changes. Longitudinal studies constitute an adequate design to evaluate the association between food consumption, oral hygiene behaviours, and dental caries. Secondly, the use of a questionnaire for caregivers may have induced a response bias, since investigators have to rely on participants’ memories to acquire information on the diet of children through their parents/guardians, as they may not remain with their children throughout the day. Therefore, the findings only refer to consumption at home. Because the number of participants in this study was large, it was decided to use a food frequency and hygiene habits questionnaire to obtain quantitative information about usual food consumption pattern and oral habits. The focus was on the frequency of breastfeeding, bottle-feeding, and snacking rather than on measuring specific dietary contents, but we tried to include some types of food and beverages or times of eating and drinking, as they are well established as a risk factor for ECC. In the present study, there were a number of short questions concerning diet, which did not perform well in terms of their relationship to a more detailed dietary assessment method. Thus, further longitudinal studies are needed to examine the interactions between specific foods and beverages and dental caries to identify the association between dietary habits and the occurrence of caries among young children. Therefore, the results should be interpreted with caution. In addition to those that have already been mentioned, the possibility of obtaining answers that do not reflect reality is another limitation. In the present study, over-reporting is a recognized limitation. Paediatricians and paediatric dentists provide parents or caregivers with instructions, and so the latter are aware of the necessity of oral health behaviours. This is a potential risk of response bias in the questionnaire reply, as it is a recognized limitation of parent-reported assessment methods in all such surveys. A notable example is the mother’s denial to the question of whether a child falls asleep during nursing (breast or bottle) and at the same time confirming feeding at night or before bedtime to calm it down. The authors of the present study encountered limitations due to lack of information on several potential important confounders. Furthermore, since ECC has numerous causes, the study did not examine all of them but focused on the factors with an important impact.

The strength of this study involves the use of both a questionnaire and a clinical examination for caries. Prevalence and severity of ECC/S-ECC were determined by clinical evaluation and were scored by experienced paediatric dentists.

## 5. Conclusions

On the basis of the results in this study, it was possible to establish the following determinants of the oral health in toddlers even with simultaneous exposure to sugar: the way a child is nourished from the moment of their birth, including breastfeeding in the first six months of life, introduction of dishes that are gradually less and less fragmented as more teeth erupt with encouragement to eat food that requires chewing, vitamin D supplementation, and parent-supervised toothbrushing from the moment the first tooth breaks through. Breastfeeding and bottle-feeding after a child is 18 months old, the reluctance to eat solids, lack of vitamin D supplementation, hygienic neglect, and delayed implementation of appropriate hygienic and dietary behaviours may contribute to the development of caries in toddlers.

## Figures and Tables

**Table 1:** The age structure of the examined children.

Age of a Child	*n* (%)	Mean Age ± SD (in Months)
12–18 months	135 (27.2%)	15.52 ± 2.06
>18–24 months	128 (25.8%)	21.76 ± 1.83
>24–30 months	129 (26.0%)	27.85 ± 1.77
>30–36 months	104 (21.0%)	33.75 ± 1.55

**Table 2:** The socio-economic characteristics of the parents and the health status of the mother of the examined children.

Parameters		*n* (%)
Total		496 (100)
Age (in years)		
Mother’s	≤25	43 (8.7)
	26 ≤ 30	164 (33.1)
	31 ≤ 35	200 (40.3)
	36 ≤ 40	77 (15.5)
	>40	12 (2.4)
Father’s	≤25	21 (4.2)
	26 ≤ 30	135 (27.2)
	31 ≤ 35	194 (39.1)
	36 ≤ 40	108 (21.8)
	>40	38 (7.7)
Education status		
Mother’s	primary and vocational	19 (3.8)
	secondary and post-secondary	157 (31.7)
	higher	320 (64.5)
Father’s	primary and vocational	16 (3.2)
	secondary and post-secondary	156 (31.5)
	higher	324 (65.3)
Economic status		
Self-assessed economic status	low	8 (1.6)
	middle	234 (47.0)
	high	254 (51.2)
Mother’s health		
Chronic diseases		126 (25.4)
Diseases during pregnancy		120 (24.2)

**Table 3:** Dietary and hygienic behaviours of children.

Parameters	*n* (%)
Breastfeeding in the first six months of life	431 (86.9)
Breastfeeding only (in the first six months of life)	174 (35.1)
Breastfeeding >12th month of life	127 (25.6)
Breastfeeding >18th month of life (100% = 391 children)	59 (15.1)
Bottle-feeding with formula >12th month of life	112 (22.6)
Bottle-feeding with formula >18th month of life (100% = 391 children)	84 (21.5)
Child’s current dietary pattern:	
>3 snacks per day	228 (46.0)
Offering fruit as snacks	475 (95.8)
Reluctance to chew food	105 (21.2)
Supplementation with vitamin D continued >12th month of life	428 (86.3)
Toothbrushing at least once a day	449 (90.5)

**Table 4:** The incidence and severity of S-ECC.

Parameters	Total	Age Groups (in Months)			
		12–18	>18–24	>24–30	>30–36
	N (%)				
Sample Size	496	105	129	133	129
S-ECC > 0 *	222 (44.8%)	20 (19.0%)	55 (42.6%)	71 (53.4%)	76 (58.9%)
	mean ± SD				
d1,2 d ≥ 3 mft **	2.62 ± 3.88	0.71 ± 1.67	2.05 ± 2.91	3.32 ± 4.28	4.02 ± 4.77
d1,2 d ≥ 3 mfs ***	4.46 ± 8.42	1.09 ± 3.07	3.80 ± 6.61	5.02 ± 7.72	7.27 ± 11.98
Number of teeth	15.40 ± 4.60	9.21 ± 3.67	14.27 ± 2.83	17.48 ± 2.73	19.40 ± 1.28

Note: * the incidence of S-ECC (S-ECC > 0); ** the severity of caries (for tooth) (d1,2 d ≥ 3 mft); *** the severity of caries (for surface) (d1,2 d ≥ 3 mfs); “d1,2”—non-cavitated carious lesions; “d ≥ 3”—cavitated carious lesions, decayed lesion; dmft—decayed, missing, filled primary teeth; dmfs—decayed, missing, filled surfaces; dt(s)—decayed tooth (surface); mt(s)—missing tooth (surface); ft(s)—filled tooth (surface).

**Table 5:** Spearman’s correlation indicating socio-economic factors and oral health behaviours related to caries.

Factors	Spearman’s Rank Correlation Coefficient (r)		
	d1,2 d ≥ 3 mft **	d1,2 d ≥ 3 mfs **	S-ECC > 0 ***
Level of education			
Mother’s	−0.290 *	−0.285 *	−0.230 *
Father’s	−0.292 *	−0.289 *	−0.231 *
Age			
Mother’s	−0.137 *	−0.138 *	−0.116 *
Father’s	−0.141 *	−0.144 *	−0.120 *
Self-assessed economic status of the family	−0.186 *	−0.177 *	−0.124 *
Vit. D supplementation > 12th month of life			
The whole sample	−0.112 *	−0.119 *	−0.119 *
Age group > 24th–30th month of life	−0.165	−0.193 *	−0.141
Breastfeeding in the first six years of life			
The whole sample	−0.088	−0.095 *	−0.087
Age group 12th–18th month of life	−0.212	−0.229	−0.221
Age group > 18th–24th month of life	−0.164	−0.186 *	−0.126
Bottle-feeding with formula only in the first six months of life			
The whole sample	0.160 *	0.164 *	0.130 *
Age group 12th–18th month of life	0.262	0.266	0.272
Age group > 18th to 24th month of life	0.182	0.213 *	0.140
Age group > 24th–30th month of life	0.228 *	0.205 *	0.165
Feeding the child > 18th month (children > 18th–36th month of life)			
Breastfeeding	0.116 *	0.103 *	0.088 *
Bottle-feeding	0.087	0.094 *	0.091 *
Age at which solids were introduced			
The whole sample	0.149 *	0.152 *	0.156 *
Age group > 18th–24th month of life	0.220 *	0.218 *	0.239 *
Number of meals per day			
The whole sample	0.276 *	0.263 *	0.185 *
Age group > 24th–30th month of life	0.408 *	0.378 *	0.201 *
Age group > 30th–36th month of life	0.358 *	0.369 *	0.255 *
Reluctance to consume foods requiring chewing			
The whole sample	0.108 *	0.106 *	0.090 *
Age group > 18th–24th month of life	0.237 *	0.236 *	0.203 *
Age group > 24th–30th month of life	0.173 *	0.170	0.173 *
Brushing the child’s teeth at least once a day			
The whole sample	−0.092 *	−0.101 *	−0.086
Age group > 30th–36th month of life	−0.176 *	−0.172	−0.168
Age when toothbrushing was introduced			
The whole sample	0.145 *	0.148 *	0.132 *
Age group > 24th–30th month of life	0.192 *	0.203 *	0.165

* Statistically significant Spearman’s rank correlation coefficients (statistical significance at *p* ≤ 0.05 based on *t*-test); ** the severity of caries (d1,2 d ≥ 3 mft/d1,2 d ≥ 3 mfs); *** the prevalence of S-ECC (S-ECC > 0); “d1,2”—non-cavitated carious lesions; “d ≥ 3”—cavitated carious lesions, decayed lesion; dmft—decayed, missing, filled primary teeth; dmfs—decayed, missing, filled surfaces; dt(s)—decayed tooth (surface); mt(s)—missing tooth (surface); ft(s)—filled tooth (surface).

**Table 6:** The incidence and severity of S-ECC.

Parameters		S-ECC > 0 **	d1,2 d ≥ 3 mfs ***
Vitamin D supplementation >12th month of life	Yes	18/60 (30.0%)	2.32 ± 5.01
	No	204/436 (46.8%)	4.75 ± 8.75
	*p*	0.014 *	*p* = 0.036 *
	OR	0.49 (0.27–0.87) *p* = 0.016 *	
	AOR−4	0.52 (0.28–0.98) *p* = 0.042 *	
Bottle-feeding exclusively with formula in the first six months of life	Yes	40/65 (61.5%)	8.66 ± 13.06
	No	181/430 (42.1%)	3.82 ± 7.30
	*p*	0.003 *	<0.001 *
	OR	OR = 2.20 (1.29–3.76) *p* = 0.004 *	
	AOR-4	OR = 1.92 (1.09–3.39) *p* = 0.024 *	
Breastfeeding only (in the first six months of life)	Yes	74/176 (42.0%)	3.65 ± 7.03
	No	148/320 (46.3%)	4.90 ± 9.08
	*p*	0.368	0.116
	OR	OR = 0.84 (0.58–1.22) *p* = 0.368	
	AOR-4	OR = 0.86 (0.58–1.27) *p* = 0.446	
Breastfeeding >18th month (100% = 391 children)	Yes	18/28 (64.3%)	6.18 ± 7.40
	No	184/363 (50.7%)	5.30 ± 9.27
	*p*	*p* = 0.166	*p* = 0.624
	OR	OR = 1.75 (0.79–3.90) *p* = 0.170	
	AOR-4	OR = 1.72 (0.70–4.25) *p* = 0.239	
Bottle-feeding with infant formula >18th month (100% = 391 children)	Yes	34/63 (54.0%)	5.98 ± 9.13
	No	168/328 (51.2%)	5.24 ± 9.16
	*p*	0.684	*p* = 0.555
	OR	OR = 1.12 (0.65–1.92) *p* = 0.689	
	AOR-4	OR = 1.27 (0.69–2.34) *p* = 0.434	
More than three snacks a day	Yes	112/228 (49.1%)	6.31 ± 10.66
	No	110/268 (41.0%)	2.88 ± 5.44
	*p*	0.071	*p* < 0.001 *
	OR	OR = 1.39 (0.97–1.98) *p* = 0.072 *	
	AOR-4	OR = 0.17 (0.08–0.36) *p* < 0.001 *	
Reluctance to consume foods that require chewing	Yes	57/105 (54.3%)	6.12 ± 10.76
	No	165/391 (42.2%)	4.01 ± 7.63
	*p*	0.027 *	*p* = 0.022 *
	OR	OR = 1.63 (1.05–2.51) *p* = 0.028 *	
	AOR-4	OR = 1.52 (0.94–2.44) *p* = 0.085	
Brushing the child’s teeth	Yes	194/451 (43.0%)	4.33 ± 8.57
	No	28/45 (62.2%)	5.73 ± 6.75
	*p*	0.014 *	*p* = 0.286
	OR	OR = 0.46 (0.24–0.86) *p* = 0.015 *	
	AOR-4	OR = 0.47 (0.24–0.93) *p* = 0.030 *	

* Wald test; *p* < 0.05; OR—odds ratio based on simple logistic regression; AOR—adjusted odds ratio based on multiple logistic regression; AOR-4 confounders—socio-economic factors, hygienic and dietary behaviours; ** the prevalence of S-ECC (S-ECC > 0); *** the severity of caries (d1,2 d ≥ 3 mft/d1,2 d ≥ 3 mfs); “d1,2”—non-cavitated carious lesions; “d ≥ 3”—cavitated carious lesions, decayed lesion; dmft—decayed, missing, filled primary teeth; dmfs—decayed, missing, filled surfaces; dt(s)—decayed tooth (surface); mt(s)—missing tooth (surface); ft(s)—filled tooth (surface).

## Data Availability

The datasets generated for this study are available on request to the corresponding authors. After results have been published, an anonymized dataset will be made publicly available at an appropriate data archive.

## Supplementary Materials

The following supporting information can be downloaded at: https://www.mdpi.com/article/10.3390/nu14204358/s1, Table S1: The incidence and severity of S-ECC based on multiple logistic regression analysis.
