# Supplementary material for: Vitamin D Supplementation, Characteristics of Mastication, and Parent-Supervised Toothbrushing as Crucial Factors in the Prevention of Caries in 12- to 36-Month-Old Children
Source: Nutrients. 2022 Oct 18;14(20):4358. doi: 10.3390/nu14204358 (PMC9607142; doi:10.3390/nu14204358)
Supplement: Supplementary file 1 [file nutrients-14-04358-s001.zip › nutrients-1945127-supplementary.pdf]

Supplemental Material

Table S1. The incidence and severity of S-ECC based on multiple logistic regression analysis.

Parameters		S-ECC > 0**
Vitamin D supplementation > 12th month of life	AOR-1	0.55 (0.30-1.01) p=0.052
	AOR-2	0.48 (0.27-0.86) p=0.014*
	AOR-3	0.49 (0.27-0.89) p=0.019*
Bottle-feeding exclusively with formula in the first six months of life	AOR-1	OR=2.12 (1.23-3.64) p=0.006*
	AOR-2	OR=2.16 (1.26-3.71) p=0.005*
	AOR-3	OR=2.01 (1.15-3.5) p=0.014*
Breastfeeding only	AOR-1	OR=0.86 (0.59-1.25) p=0.434
(in the first six months of life)	AOR-2	OR=0.84 (0.57-1.21) p=0.345
	AOR-3	OR=0.85 (0.58-1.25) p=0.407
Breastfeeding > 18th month (100% = 391 children)	AOR-1	OR=1.51 (0.64-3.52) p=0.344
	AOR-2	OR=1.72 (0.77-3.86) p=0.186
	AOR-3	OR=2.06 (0.89-4.76) p=0.091
Bottle-feeding with infant formula >18th month	AOR-1	OR=1.24 (0.70-2.19) p=0.455
(100% = 391 children)	AOR-2	OR=1.21 (0.70-2.09) p=0.487
	AOR-3	OR=0.99 (0.56-1.77) p=0.982
More than three snacks a day	AOR-1	OR=1.33 (0.92-1.92) p=0.134
	AOR-2	OR=1.36 (0.95-1.95) p=0.089
	AOR-3	OR=0.17 (0.08-0.34) p<0.001*
Reluctance to consume foods that require	AOR-1	OR=1.57 (1.00-2.46) p=0.051
chewing	AOR-2	OR=1.64 (1.06-2.54) p=0.025*
	AOR-3	OR=1.55 (0.98-2.45) p=0.060
Brushing the child's teeth	AOR-1	OR=0.43 (0.22-0.84) p=0.013*
	AOR-2	OR=0.46 (0.24-0.86) p=0.015*
	AOR-3	OR=0.49 (0.25-0.94) p=0.033*

AOR-1 adjusted odds ratio for the first multivariate model where confounder are socio-economic factors, AOR-2 confounders: hygienic behaviours, AOR-3 confounders: dietary behaviours. * Wald test; $p < 0.05$. ** the prevalence of S-ECC (S-ECC > 0).
